# Managing risk and resilience in autonomous and intelligent systems: Exploring safety in the development, deployment, and use of artificial intelligence in healthcare

**DOI:** 10.1111/risa.14273

**Published:** 2024-01-21

**Authors:** Carl Macrae

**Affiliations:** ^1^ Nottingham University Business School, University of Nottingham Nottingham UK; ^2^ School of Health and Welfare Halmstad University Halmstad Sweden

**Keywords:** artificial intelligence, resilience, risk regulation, sociotechnical risk

## Abstract

Autonomous and intelligent systems (AIS) are being developed and deployed across a wide range of sectors and encompass a variety of technologies designed to engage in different forms of independent reasoning and self‐directed behavior. These technologies may bring considerable benefits to society but also pose a range of risk management challenges, particularly when deployed in safety‐critical sectors where complex interactions between human, social, and technical processes underpin safety and resilience. Healthcare is one safety‐critical sector at the forefront of efforts to develop and deploy intelligent technologies, such as through artificial intelligence (AI) systems intended to automate key aspects of healthcare tasks such as reading medical images to identify signs of pathology. This article develops a qualitative analysis of the sociotechnical sources of risk and resilience associated with the development, deployment, and use of AI in healthcare, drawing on 40 in‐depth interviews with participants involved in the development, management, and regulation of AI. Qualitative template analysis is used to examine sociotechnical sources of risk and resilience, drawing on and elaborating Macrae's (2022, *Risk Analysis*, *42*(9), 1999–2025) SOTEC framework that integrates structural, organizational, technological, epistemic, and cultural sources of risk in AIS. This analysis explores an array of sociotechnical sources of risk associated with the development, deployment, and use of AI in healthcare and identifies an array of sociotechnical patterns of resilience that may counter those risks. In doing so, the SOTEC framework is elaborated and translated to define key sources of both risk and resilience in AIS.

## 1 INTRODUCTION

Efforts to develop and deploy autonomous and intelligent systems (AIS) have exploded in recent years, from self‐driving cars to healthcare chatbots. AIS are technologies that engage in some form of independent reasoning and self‐directed behavior: perceiving, predicting, planning, and performing in relation to some particular problem (e.g., diagnosing a health condition) or environment (e.g., navigating public roads) through the application of artificial intelligence (AI). These technologies have the potential to transform industries and may bring substantial benefits to society, but they also pose considerable risk. This is particularly the case in safety‐critical arenas such as healthcare and transport, where AI has the potential to create new risks, or amplify or transform existing risks, in ways that are not well understood—illustrated by fatal failures of intelligent systems in transport (NTSB, 2019) and increasing political attention on AI safety (UK Prime Minister's Office, 2023).

Healthcare is at the forefront of attempts to develop and deploy AI, including efforts to support or replace clinical diagnosis (De Fauw et al., 2018), engage with and assist patients (Babic et al., 2021), and optimize treatment planning and clinical services (Kearney et al., 2018). These efforts are particularly advanced in relation to clinical tasks amenable to machine learning, such as reading medical images in radiology and ophthalmology (Ranschaert et al., 2019; Zhou et al., 2023) or augmenting visual detection of tumors in gastroenterology (Wallace et al., 2022). Several hundred AI and machine learning–enabled medical devices are already registered with regulators (FDA, 2022), and the use of AI has been recommended by national bodies for specific tasks such as targeting radiotherapy (NICE, 2023). An enormous amount of work is currently underway to understand how to safely deploy, integrate, use, and manage AI in different healthcare settings (CQC, 2020; Feng et al., 2022; Sendak, 2020; Svedberg et al., 2022; Wiens et al., 2019; Winter & Carusi, 2023).

Safely deploying and managing AI in healthcare presents a range of challenges that span from the “sharp end” of care to the “blunt end” of regulation. These include human factors challenges associated with integrating intelligent technologies into complex and dynamic processes of healthcare (Nilsen et al., 2022; Smith et al., 2021); social and cultural challenges of understanding and adapting to technologies that displace or transform work previously done by humans (Sujan et al., 2019); and organizational and regulatory challenges of monitoring and assuring the safety of new technologies that operate in novel and unexpected ways (Larson et al., 2021; Wiens et al., 2019). Moreover, it is broadly accepted that the safety of healthcare depends on highly adaptive and resilient human performance that can accommodate the inherent disruptions, uncertainties, and variabilities in activities of care (Anderson et al., 2020; Lyng et al., 2022; Macrae & Draycott, 2019). Introducing complex intelligent technologies into these already complex and dynamic settings is therefore likely to pose many risk management challenges.

**FIGURE 1:**
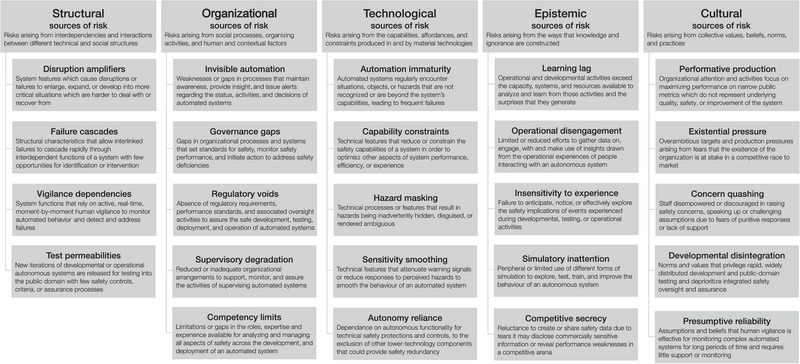
The structural, organizational, technological, epistemic and cultural (SOTEC) framework of sociotechnical sources of risk in autonomous and intelligent systems (from Macrae, 2022).

This article explores the sociotechnical sources of risk and resilience associated with the development, deployment, and use of AI in healthcare. Decades of sophisticated sociotechnical analysis have produced a range of theoretical perspectives which explain the complex interplay between human, social, and technical factors in the emergence and management of risk. This rich body of work approaches sociotechnical risk from a variety of perspectives, examining system characteristics and structures that can render complex systems particularly vulnerable to catastrophic breakdown (e.g., Perrow, 1999), social and cultural practices that can allow failures to incubate and enlarge (Turner, 1976, 1978; Vaughan, 1996), and human and organizational processes that can defeat risk controls and safety defenses (e.g., Reason, 1997). These diverse perspectives have been brought together to explain the nature of sociotechnical risk associated with AI in the SOTEC framework (Macrae, 2022) which characterizes structural, organizational, technological, epistemic, and cultural sources of risk (Box 1 and Figure 1). However, the range of sociotechnical sources of risk and safety associated with AI in healthcare have yet to be systematically explored (Macrae, 2019).

Accordingly, the overarching aims of this article are to apply, develop, and extend the SOTEC framework to characterize the sociotechnical sources of risk associated with AI in healthcare, and identify principles of sociotechnical resilience that can counter those risks and which may be applicable to AIS more generally. To do this, the article presents an analysis of a set of in‐depth interviews with participants active in the field of healthcare AI, including technology developers, clinicians, healthcare managers, and regulators. Throughout this analysis AI is defined broadly, as any technology that is able to engage in some form of independent reasoning and self‐directed behavior without the need for real‐time human input. In the context of healthcare, this commonly takes the form of medical devices or software applications involved in interpreting medical data (such as images, videos, scans, or electronic health records, e.g., Xie et al., 2020) to identify or diagnose certain health conditions (e.g., abnormalities in a chest X‐ray) or plan or recommend some future action (e.g., indicate a decision to refer or discharge a patient). However, given the rapid pace of innovation and the wide range of emerging AI applications, this study purposefully takes a broad approach to explore a wide range of current and potential sources of risk and resilience. Similarly, healthcare is a highly varied and complex sector that encompasses an enormous range of services, professionals, and organizations that are involved in providing a huge range of care to people in different settings. In this study, healthcare is considered broadly, but the primary focus is on care that is provided in complex organizational settings such as hospitals or other clinics, where care is typically managed and delivered by a variety of specialist healthcare professionals and allied health workers. Through this analysis, specific instances and exemplars of sociotechnical risk are explored, new patterns of sociotechnical risk are defined, and sociotechnical sources of resilience are identified across each of the structural, organizational, technological, epistemic, and cultural domains of the SOTEC framework.

**BOX 1. Summary and background of the SOTEC framework of sociotechnical sources of risk in autonomous and intelligent systems**

**The SOTEC framework of sociotechnical risk in autonomous and intelligent systems**

**SOTEC background**

The SOTEC analysis framework (Macrae, 2022) was developed to define and characterise fundamental sociotechnical sources of risk in autonomous and intelligent systems (AIS). AIS are necessarily designed, developed and deployed in complex social and organizational contexts and many of the risks associated with these systems therefore arise in the sociotechnical interactions that unfold around those processes. The SOTEC framework was developed through a systematic analysis of one of the most publicly examined failures of AIS: the fatal Uber self‐driving car collision in Tempe, Arizona in 2018 (NTSB, 2019). An extensive re‐analysis of that event was grounded in empirical analysis of 48 in‐depth public reports into the event, including both government and corporate investigations and investigative media reporting. The analysis was theoretically oriented and sought to integrate and extent existing theories of sociotechnical risk (e.g. Perrow, 1984; Downer, 2011; Macrae, 2014; Reason, 1997; Turner, 1976; Vaughan, 1996).

**SOTEC summary**

The SOTEC analysis framework identifies five fundamental and interrelated sources of sociotechnical risk: structural, organizational, technological, epistemic and cultural. Each of these domains is characterised by distinct patterns of sociotechnical failure, and each of these five domains of risk draws on a particular theoretical lineage and offers a complementary conceptual lens through which to explore and explain the sociotechnical sources of risk in AIS:

- *Structural* sources of risk arise from interdependencies and interactions between different parts of the social and technical structures that constitute AIS, such as ‘failure cascades’, where structural characteristics allow interlinked failures to cascade rapidly through interdependent functions of a system with few opportunities for identification or intervention;
- *Organizational* sources of risk arise from the social processes, organizing activities and human and contextual factors that underpin AIS, such as ‘invisible automation’, where there are weaknesses or gaps in processes that maintain awareness, provide insight and issues alerts regarding the status, activities and decisions of highly automated systems;
- *Technological* sources of risk arise from the capabilities, affordances and constraints inscribed into and produced by the material technologies of AIS, such as ‘hazard masking’, where technical processes or features result in hazards being inadvertently hidden, disguised or rendered ambiguous;
- *Epistemic* sources of risk arise from the ways that knowledge and ignorance are constructed in relation to, and within, AIS such as ‘learning lag’, where operational and developmental activities exceed the capacity, systems and resources available to analyse and learn from those activities and the surprises that they generate; and
- *Cultural* sources of risk arise from the collective values, beliefs, norms and practices that surround and shape AIS, such as ‘performative production’, where organisational attention and activities focus on maximising performance on narrow public metrics which do not represent underlying quality, safety or improvement of a system (Macrae, 2022).

## 2 METHODS AND APPROACH

The primary objective of this analysis is to characterize sociotechnical sources of risk associated with the development, deployment, and use of AI in healthcare and identify associated sociotechnical principles of resilience that may counter those risks. The secondary objective is to elaborate and develop the SOTEC framework, to both contextualize it within the domain of healthcare and to extend it to encompass “positive” sources of resilience as well as “negative” sources of risk. As indicated above, in this study AI was explored expansively in order to enable the development of a widely‐applicable analytical framework. Here, AI was defined as encompassing any innovative digital technology that could engage in some form of independent analysis, reasoning, or decision making that supports the delivery of healthcare, and that typically (but not necessarily) involves the processing of large quantities of healthcare data enabled by machine learning algorithms. This approach purposefully spans a wide range of technologies and applications, though some of the most advanced and widely deployed AI applications in healthcare currently focus on tasks associated with the clinical analysis, interpretation, and reporting of medical images (such as scans of the retina, e.g., Xie et al., 2020), and these types of application were a common reference point throughout the study.

A diverse group of stakeholders involved in the development, deployment, management, and regulation of AI was engaged for the same reason. AI safety is a rapidly advancing field, with many diverse actors exploring and encountering an expanding range of risks, and new safety strategies being actively developed. In healthcare, key types of stakeholder involved in these activities include technology developers, clinicians, regulators, healthcare managers, public and patient representatives, and other subject matter experts. Given the relative novelty and rapid emergence of many AI technologies, there is little widely agreed guidance on AI risk management and safety assurance in healthcare, and many of the fundamental risk and safety issues remain the focus of active policy debate and practical exploration. The methodological approach adopted here was therefore qualitative and oriented to conceptual development, based on an interview study designed to engage with a diverse group of participants who were all actively involved in the deployment, use, governance, regulation, and safety analysis of AI in healthcare. Semistructured interviews were used to explore these participants’ practical experiences and perspectives on the nature of risk and resilience and the management of safety in relation to AI. The underlying analytical approach was qualitative and oriented to conceptual development, with a particular focus on the empirical exploration and elaboration of the patterns of sociotechnical risk characterized in the SOTEC framework (Macrae, 2022). Accordingly, interview data were analyzed using template analysis (King, 2004), a systematic method for analyzing qualitative data in relation to a predefined coding template. The SOTEC framework provided the initial coding template that was elaborated and built on through this analysis. The study received favorable ethical review by Nottingham University Business School Research Ethics Committee, and interviews were carried out between August 2019 and August 2021.

### 2.1 Sampling and recruitment

A diverse group of participants was purposefully sampled based on participants’ practical experience and active involvement in the work of developing, deploying, governing, regulating, and analyzing AI systems in the English healthcare system. These participants were primarily identified through prominent public and professional activities (e.g., high‐profile healthcare organizations doing advanced work in AI); through their involvement in exploratory public regulatory activities (e.g., regulatory engagement and “sandboxing” activities examining the use of AI); and through associated snowball sampling (Parker et al., 2019) through professional referral of additional key stakeholders active in the field. A key recruitment criteria was that all participants had to be actively and practically involved in the management, development, regulation, implementation, or analysis of new intelligent technologies in healthcare, in order that interview discussions could be based on practical insight and concrete experience, and not simply speculation or conjecture.

Once identified as a potential participant, individuals were approached via email and provided with information about the research along with a request to participate. When participants agreed to participate, each was provided with documentation regarding consent to participate, including details on how data would be used and that identities of participants would remain undisclosed to ensure anonymity and encourage open and honest interview discussions. Consent forms were signed by both interviewee and interviewer to formalize this agreement prior to interviews taking place.

A total of 35 participants were interviewed (Table 1), selected to provide a diverse range of perspectives and a deep set of practical insight and experience into the challenges of managing safe AI in healthcare. Around half of these participants (*n* = 17) were selected based on their active regulatory or national governance role in relation to AI in the English healthcare system. These participants were drawn from national standard setting bodies, regulators of care provider organizations, regulators of medical devices, and national health policy agencies and were all directly involved in the development of policy and standards, or the regulatory inspection, assessment, certification, or review of AI systems and associated care processes. The other 18 participants were selected to provide insight from healthcare organizations, technology developers, and subject matter experts. This included seven participants from technology companies producing and marketing healthcare AI systems, represented by participants with compliance, governance, technical development, and leadership roles. It also included nine participants from healthcare organizations, encompassing six clinicians working in the field of AI development and deployment, health informatics and clinical safety (with background specialties including radiology, ophthalmology, and pediatrics), and three healthcare managers with responsibility for deploying, managing, and governing AI technologies. And it included two domain experts: a human factors expert working in healthcare AI, and an “expert by experience” representative of patients working with a regulator on AI issues.

**TABLE 1:** Interview participants.

Participants, type of participation, and interviews conducted			
Participation type	Participants	Interviews	Description
*Regulation participants*			
Regulators (R)^a^	17	22^b^	Regulators and standard‐setters for healthcare providers, medical devices, clinical technologies, and evidence.
*Health sector participants*			
Technology developers (T)	7	7	Developers and manufacturers of artificeal intelligence (AI) technologies including compliance and clinical leadership.
Clinicians (C)	6	6	Clinicians involved in patient care, clinical research, and AI implementation and evaluation.
Healthcare managers (M)	3	3	Health system managers involved in commissioning, implementation, evaluation of clinical AI.
Domain experts (E)	2	2	Subject matter experts or experts‐by‐experience involved in analysis of AI safety and implementation in health.
*Subtotal*	*18*	*18*	
**Total**	**35**	**40**	

^a^ Capital letters in parenthesis are used in the analysis section to indicate the source of relevant data excerpts, with the interview number appended to maintain anonymity of participants. For instance, (R07) would indicate the source of a quote was a participant who was a regulator and the quote was from interview number 7 of the 40 conducted.

^b^ Of the 17 regulators who participated, five were interviewed twice over a 9‐month period due to their intensive involvement in ongoing and evolving activities of AI regulation and policy development.

### 2.2 Data collection and analysis

Data were collected through semistructured interviews with the 35 participants. A total of 40 interviews were conducted, each between 30 and 60 minutes in length. Five of the participants who were actively involved in a program of work developing regulatory standards for AI safety were interviewed twice over a 9‐month period (2019–2020) to more deeply explore their developing insights and the evolving regulatory approach that was being developed. A topic guide (Box 2) was followed in all interviews that explored six key areas of participants’ experiences of AI safety in healthcare. Interview questions explored: (1) the safety benefits of applying AI in healthcare; (2) the safety challenges associated with introducing AI technologies in healthcare; (3) the organizational and practical changes associated with introducing AI in healthcare; (4) the governance systems and regulatory requirements suitable for AI in healthcare; (5) the organizational processes required to respond to AI system failures; and (6) what is needed to ensure trustworthy AI in healthcare.

**BOX 2. Semi‐structured interview topic guide**

**Semi‐structured interviews: topic guide**

1. What do you see as the main benefits of applying AI/ML in healthcare?
  1. How do you think these technologies might help to improve patient safety?
  2. What are the most promising and beneficial applications?
2. What would you see as the key safety challenges that might be introduced with AI/ML technologies?
  1. How might these technologies experience problems and how could this impact on patients and potentially cause harm?
  2. What would be your biggest safety concerns of applying these technologies in future?
  3. How well do we understand the risks and benefits of implementing AI/ML in healthcare at the moment?
  4. What more do we need to find out?
3. How do you expect AI/ML technologies to change the way work is done in [this / your] setting?
4. To what extent do recent regulatory and policy activities address the key safety issues associated with AI/ML applications?
  1. In your view, what sorts of oversight and regulation might be needed to assure the safety of AI/ML technologies in healthcare?
  2. To what extent are current governance and safety systems appropriate?
  3. In what ways might they need to be changed?
5. What do you think should happen if an AI/ML system was implicated in a patient safety event?
6. What is needed to build trust in AI/ML technologies amongst clinicians and other professionals?
  1. What is needed to build trust amongst patients, families and the public?
  2. What sort of evidence or disclosures might be required?

**FIGURE 2:**
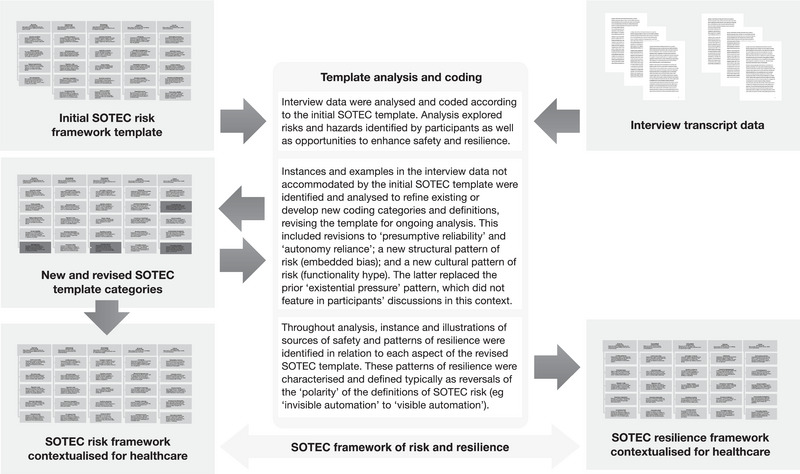
Template analysis and coding process.

**FIGURE 3:**
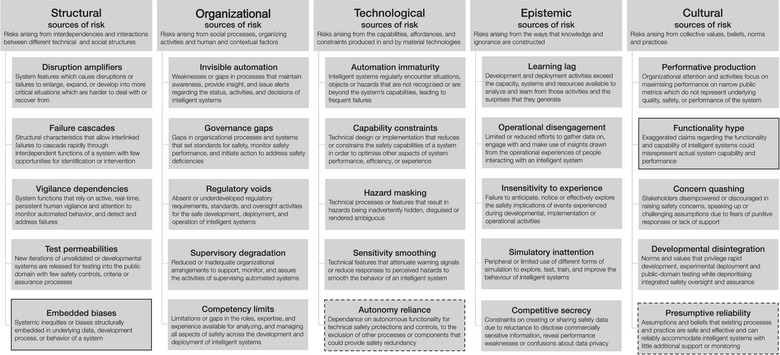
Extended and contextualized structural, organizational, technological, epistemic and cultural (SOTEC) framework of the sociotechnical patterns of risk associated with developing, deploying, and using artificial intelligence in healthcare. *Note*. Dotted outline indicates substantively updated category; full outline indicates new category.

Interviews were transcribed and qualitative data analysis was conducted in line with the principles of template analysis (King, 2004), using the SOTEC framework as the initial coding template. In the first instance, all interview transcripts were systematically reviewed and all relevant instances and exemplars of sociotechnical patterns of risk and resilience were identified, labeled, and coded, with particular reference to the SOTEC framework (Figure 2). This coding process involved identifying instances in the interview data that illustrated, contextualized, or extended a particular category of the SOTEC framework, while remaining sensitive to both the “negative” sources of risk associated with AI systems as well as the “positive” sources of resilience that may enhance AI safety. Where instances and exemplars were identified that were not fully accommodated by any of the existing SOTEC framework categories, new coding categories were developed and defined to extend the coding template (i.e., the SOTEC framework). For example, issues of bias and fairness were of prominent concern in relation to many AI systems in healthcare, and this was not captured in the initial SOTEC framework categories that were derived from a transport‐related analysis. Accordingly, new categories were developed and incorporated (“embedded biases” and “embedded fairness”). Similarly, several categories in the initial SOTEC framework were updated and revised. This included defining a new category of “functionality hype” as a cultural source of risk. In this analysis, that new category took the place of the prior category of “existential pressure,” because no interview data in this study and setting indicated that to be a prominent issue. Furthermore, the concept of *presumptive reliability* was expanded to include a broader range of cultural assumptions about the safety of existing processes and practices, beyond the initial focus on real‐time human supervisory monitoring (a primary concern in vehicle operation but less so in many healthcare settings). And the concept of *autonomy reliance* was extended to encompass the risks of relying on autonomous functionality to the exclusion of a much wider range of safety controls and protective mechanisms, extending beyond the purely technical redundancies that are often a key source of resilience in transport systems. As a result of this analysis, two new patterns of sociotechnical risk were characterized, two were substantively revised (see Figure 3), and positive sources of resilience were derived for all SOTEC concepts.

## 3 FINDINGS AND ANALYSIS: RISK, RESILIENCE, AND AI IN HEALTHCARE

Participants involved in developing, deploying, and managing AI in healthcare articulated a wide range of concerns regarding the risks associated with these new technologies. They also indicated a set of associated ideals for how safety and resilience should be supported to counter these risks. This enabled the contextualization and elaboration of the five domains of the SOTEC framework (Figure 3). These structural, organizational, technological, epistemic, and cultural sources of risk and resilience are examined in turn.

### 3.1 Structural sources of risk and resilience

Structural sources of risk and resilience arise from interdependencies and interactions that emerge between the different technical and social structures that surround and support intelligent systems. Structural issues underpinned a range of concerns and opportunities for participants.

#### 3.1.1 Disruption amplifiers and disruption mitigators

Participants were concerned about *disruption amplifiers* introduced by AI into healthcare systems: structural features which can cause disruptions or failures to enlarge or develop into more critical situations that are harder to recover from. This could involve systematically missing serious clinical conditions in triaging or screening processes, where AI is intended to automatically deprioritize or remove cases with apparently normal clinical signs: “the risk is that you identify a ‘normal’ and no one cares any more. And all it takes is missing one case and the patient turns up 6 months later with something that was obviously there” (M08). This was particularly hazardous in relation to serious and time‐critical diagnoses, where “there may be a narrow window when you can intervene, and a large treatment effect, so the cost of a false negative could be someone's life” (C10). Some clinicians emphasized the potential to implement AI in ways that mitigated such disruptions by limiting its application to conditions where “the [diagnostic] window isn't so small, and you have multiple opportunities” (C10) to detect problems. This indicates the potential value of AI being used in ways that create and enhance *disruption mitigators*: system features that help capture and constrain disruptions and transform them into less critical situations that are easier to deal.

#### 3.1.2 Failure cascades and failure isolators

Participants were wary of AI creating the potential for *failure cascades*, structural characteristics that allow problems to rapidly spread and cascade through a system of care with limited opportunities to intervene. Significant concerns related to the far‐reaching impacts for a care pathway if a critical AI system suddenly became unavailable due to a major technical failure or the company supporting it shutting down. Participants highlighted that AI systems are highly specialized and integrated into specific clinical settings, and care pathways are extensively redesigned around specific AI functionalities—such as providing results from medical scans within seconds while patients are still in the room, rather than several weeks later. It is therefore not straightforward to simply substitute one AI system for another if it becomes unavailable, and AI failures would have the potential to cause rapid knock‐on effects throughout entire care pathways. Participants emphasized the need to deploy clinical AI in tightly controlled ways performing clearly specified functions that can be subject to routine auditing and review of safety performance: “as this [AI] is rolled out it has to have a very, very specific function” (R22). This indicates the importance of creating *failure isolators* in the deployment and use of AI: structural features that enable the identification of failure and prevent failures cascading rapidly and irrecoverably into interdependent parts of a system.

#### 3.1.3 Vigilance dependencies and vigilance augmentations

Participants had concerns that AI could create new requirements for challenging cognitive work, introducing *vigilance dependencies* in which system functions demand persistently high levels of human attention and vigilance. This could take the form of processes that alter and increase the cognitive load on clinicians, such as requirements to individually cross‐check areas of chest X‐rays highlighted by an AI system. It could also involve increased requirements to remain vigilant against potential distractions or extraneous information from AI systems. For instance, in triaging processes “if it [AI] clutters my ‘hot list’ with junk then I don't want it” (C04). Instead, participants emphasized the opportunities for AI to be deployed in ways that supported human cognition, for instance by providing additional background checks of human clinical reviews of medical scans to check for errors: “One thing I wanted out of AI was just a hand to help spot the errors which is what started this off for me” (M08). This indicated the potential for *vigilance augmentations*: using AI to support and augment active, real‐time human attention and cognition by monitoring both human and automated behavior, and assist in detecting and addressing failures.

#### 3.1.4 Test permeabilities and test enclosures

Testing and validation of AI in healthcare was a major focus for participants, who were highly cautious of *test permeabilities*, where unvalidated and developmental systems are tested on the public with few safety controls. Considerable attention was given to the ways that AI should be safely tested and validated in clinical settings while ensuring that unvalidated systems are separated from clinical care. This included the “shadow” operation of AI systems, acting as “ghost third readers” of medical scans for prolonged periods to allow careful examination of performance, as well as “regulatory sandboxes” creating a protected space for exploring risks and regulatory requirements (CQC, 2020). One manager likened the caution needed for testing clinical AI to the early days of the motor car, where someone walked ahead of a vehicle waving a red flag. Participants emphasized the importance of tightly controlled “real world” testing, because the actual clinical performance of AI could differ from research settings: “when the algorithm is placed into real clinical practice, you can expect it to be pretty different in those situations” (C10). This indicates the principle of creating robust *test enclosures* in AI implementation: protected spaces in which AI can be tested in carefully controlled and defined arenas separated from the public.

#### 3.1.5 Embedded biases and embedded fairness

Participants had extensive concerns about *embedded biases* in AI, where systemic inequities or biases are structurally embedded in the underlying data, development, or behavior of an intelligent system. There were broad‐based concerns that AI might codify and perpetuate systemic biases and preexisting inequalities in ways that were not well understood, with considerable impacts on the care received by different groups. A core concern was bias in data sets used to develop AI: “what we are very wary of, we are programming lots of machines based on historical data… but in healthcare that could mean a whole way of working that is biased… based on how we used to do things rather than how we want to do things” (R36). Biases in current care processes also posed fundamental challenges, as those care processes provided both comparative benchmarks for AI performance, and generated the clinical data sets used to develop AI. Participants emphasized it was critical to identify and account for these biases, to “make sure that you train the algorithm on properly representative datasets, and that limitations and biases in datasets are understood and can be accounted for” (R19). This indicates the important principle of *embedded fairness*: creating and maintaining structures that embed fairness and assure and account for equity in the data, development, and deployment of intelligent systems.

### 3.2 Organizational sources of risk and resilience

Organizational sources of risk and resilience arise from the social processes, organizing activities, and human and contextual factors that surround and support intelligent systems. Organizational issues animated a range of concerns and opportunities for participants.

#### 3.2.1 Invisible automation and visible automation

Participants described a range of challenges related to *invisible automation*—weaknesses or gaps in processes needed to maintain awareness and provide insight into the status, activities, and decisions of an intelligent system. Several participants emphasized the importance of deploying AI in ways that best support human decision making, so that human and machine can “work alongside” (R22) each other. AI was expected to be carefully integrated into the working activities of professionals and clinical teams: “professionals will work in teams with these systems” (E05), which another participant described as “human machine teaming, so both are working together” (T16). To support this expected close integration of human and machine intelligence, several participants highlighted the need for processes that allowed AI to “make transparent its current state and current reasoning” (E05), and that it was increasingly important to understand “what sort of interpretability a system needs to have to be a useful partner in a decision making process” (T16). This indicated the need for *visible automation*: the development of robust and diverse processes that can enable insight and maintain awareness into the status, activities, and reasoning of intelligent systems.

#### 3.2.2 Governance gaps and governance systems

An important focus for participants was the potential for *governance gaps* to emerge around AI, where organizational processes involved in setting standards, monitoring performance, and addressing deficiencies in safety are underdeveloped or missing key elements. As one regulator described, much of their core focus concerns “what governance was put in place to ensure that they maintain standards? How have organisations made sure that they engaged with staff and users to make sure they use it [AI] well? What is the organisation's risk management plan and monitoring around what happens when things go wrong? And how do they measure improvement over time?” (R15). These considerations were central to how participants thought about managing AI safety, and pointed to the importance of developing appropriate *governance systems* alongside the technological systems of AI: robust organizational processes that set standards for safety, monitor safety performance, and initiate and organize action to address safety deficiencies.

#### 3.2.3 Regulatory voids and regulatory infrastructure

Participants highlighted risks that could arise from *regulatory voids*, where regulatory requirements or oversight activities were absent or underdeveloped in relation to AI safety. Participants were concerned that these were particularly associated with the space between regulatory approval of a particular AI‐based product or device, and its practical deployment and use in specific settings. As one regulator indicated, the gap may be where, “you've got something which you've shown is fine and accurate and tested on loads of data, but then it is the more human part around implementing it” (R30). This was highlighted in terms of developing regulatory clarity and standards around “what else needs to be included within that supported environment” (T16), and “how it [AI] is actually deployed in services” (R18). This indicates deeper concerns with building an integrated *regulatory infrastructure* to support AI safety: regulatory requirements, performance standards, and associated oversight activities that can assure the safety of AI across the full range of development, testing, deployment, and use.

#### 3.2.4 Supervisory degradation and supervisory support

Participants were mindful of *supervisory degradation*: inadequate organizational arrangements to support and assure the work of supervising automated systems. Active human oversight of AI was viewed as essential, particularly given the relatively constrained tasks AI can reliably perform: “you need a proper process check, the machine will only know what it knows” (R13). The organizational arrangements needed to support effective supervision of AI included basic education and training that should be as fundamental as, “how to read an x‐ray; how to read an AI” (C04). It included organizational systems that enable clinicians to challenge and override AI outputs: “you've got to have the ability for clinicians to override things or put in additional information” (E27). Some participants indicated that this may require a fundamental reexamination of clinical work, acknowledging the different demands of supervising automated systems compared to interacting with simpler technologies: “there is a distinction between how to use a device versus how to supervise a system” (E05). All of this indicates the importance of developing appropriate capacities for *supervisory support* around intelligent systems: organizational arrangements to support, monitor, and assure the activities of supervising intelligent systems.

#### 3.2.5 Competency limits and competency requisites

Participants were concerned about *competency limits*: limitations or gaps in the roles, expertise and experience available for managing the safety of AI. Participants believed broad‐based improvements in knowledge and skills were needed to safely purchase, deploy, and integrate AI into healthcare: “the biggest area of concern is skilling‐up the NHS [National Health Service] so it's in position where we can specify and procure these technologies… and then being able to integrate that into care processes” (R39). New dedicated roles were required. Some clinicians described themselves as developing new hybrid professional identities: “My principle role is to be a bridge between the clinical side and the technical side… I see myself as an AI medical designer” (C04). Other roles would be needed, like an “‘AI nurse’ as a new role, to supervise these systems” (E05). A priority was matching the expertise and competency available for developing AI technologies with similar expertise and competency to implement, deploy, and use those systems: “regulation needs to change so the same amount of rigour that goes into development of a product goes into the amount of rigour for a new deployment, at the end of the day if not then clinical risk will manifest in the deployment organisation” (R39). This indicates the central principle of *competency requisites*, clearly established roles and requisite expertise for analyzing and managing all aspects of safety across the development, deployment, and use of intelligent systems.

### 3.3 Technological sources of risk and resilience

Technological sources of risk and resilience arise from the capabilities, affordances, and constraints produced in and by the material technologies that constitute intelligent systems. Technological issues characterized a range of concerns and opportunities for participants.

#### 3.3.1 Automation immaturity and automation maturity

Participants were concerned about *automation immaturity*, where intelligent systems encounter situations or inputs beyond current system capabilities leading to frequent failures. Participants’ concerns encompassed fundamental technological weaknesses, such as the fragility of neural nets and the surprising mistakes these can make in response to tiny changes of input imperceptible to humans: “we might not understand why the network makes mistakes, if one pixel is out of place” (T03). One participant with lived experience of care emphasized the inherent challenges of clinical variability and rarity: “Mine [clinical condition] is so unusual, even a consultant with 30–40 years experience has never seen one in his life. Obviously with an algorithm, don't imagine it would pick up anything different [with] those rarity of issues” (E27). Another concern was whether the mature performance of clinical AI in one setting can be transferred to another and how rapidly algorithmic performance might decline: “once trained, how extensible is it? What is the point at which you apply it to a different population… what is the point when the algorithm starts to decay? How do you detect and respond and deal with that? Big generalisation questions” (R19). This all indicates an important principle of managing and monitoring *automation maturity*: assuring that intelligent systems are regularly encountering situations, objects, or hazards that are recognized and within system capabilities.

#### 3.3.2 Capability constraints and capability intensifiers

Participants were attentive to *capability constraints*, where technical design or deployment decisions might reduce or constrain safety capabilities in order to optimize other aspects of performance. Many concerns related to the motivation for deploying AI. Participants noted AI may be used to increase financial efficiencies, “using AI to cut corners, automating through ML [machine learning], so that hospitals… can make decisions that impact health and cut costs: fewer humans” (T03). Tensions between safety and efficiency were illustrated by the potential uses of AI for reading medical images: these could either take the place of a radiologist, helping to reduce staffing pressures and backlogs; or they could be implemented to increase reliability and safety by providing an additional check of human‐read images. Those close to front‐line care particularly emphasized the importance of using AI to enhance and improve safety, rather than simply increase efficiency, with one highlighting that, “my initial motivation was to use the system to find errors” (M08). This indicates the importance of using AI to create *capability intensifiers*: technological features that strengthen or extend the safety capabilities of a system as well as other aspects of system performance.

#### 3.3.3 Hazard masking and hazard highlighting

Processes of *hazard masking*—where technical processes or features can result in hazards being inadvertently hidden, disguised, or made ambiguous—were of concern. Participants noted that common AI technologies, such as neural networks, can produce incorrect predictions with high‐confidence. One technologist explained, “neural nets are easily fooled… people say ‘if the confidence is low then fail‐safe to a human’—but they [neural nets] can be highly confident *and* wrong” (T03). Relying on measures of confidence to identify the need for human intervention may therefore hide errors. Participants were also cautious about hidden limitations of AI. If a system reports it has not identified an abnormality in a medical image, it does not necessarily mean the scan is normal, because there may be abnormalities present that are outside of the range of those the system is designed to detect. One regulator emphasized the importance of understanding that, for instance: “it can have 100 things it recognises as abnormal, but it doesn't recognise ‘normal’” (R36). So, if an AI system is only capable of detecting a subset of problems, its outputs should make this clear. This indicates the importance of developing and deploying AI in ways that enhance capabilities of *hazard highlighting*, where technical processes or features enable potential hazards to be reliably highlighted or their potential existence rendered transparent.

#### 3.3.4 Sensitivity smoothing and sensitivity fidelity

Problems of *sensitivity smoothing* concerned participants, where intelligent systems incorporate technical features that attenuate warning signals or reduce responses to perceived hazards, to smooth the behavior of a system. Participants were mindful that the outputs of AI systems in healthcare—such as reporting whether abnormalities have been detected in a medical image—are determined by the thresholds and criteria set by the people designing and deploying the system. As one healthcare manager emphasized, algorithms “give you a likelihood if something is present or not, and it's up to humans to define that threshold and define that evidence, so the decision‐making for defining that threshold needs to be very clear so people understand what the system is giving them. Reducing down to a yes or no is a binary decision of convenience, it's not the reality” (M08). It is therefore critically important to be careful and rigorous in designing key thresholds, criteria, and other parameters that are embedded into intelligent systems. This indicates the importance of establishing *sensitivity fidelity* in intelligent systems by maintaining technological processes that detect and amplify warning signals appropriately, and proportionally match the response of an intelligent system to the level of perceived hazard.

#### 3.3.5 Autonomy reliance and autonomous redundancy

Participants worried about *autonomy reliance*, a dependence on autonomous functionality for safety protections and controls, to the exclusion of other processes or components that could provide safety redundancy. This was considered a risk particularly in relation to situations with no human oversight or review of AI decisions, such as the use of AI to conduct autonomous “hard” triaging of X‐rays, where an AI independently removes patients from a radiology list if no abnormalities are detected in the X‐ray films, without a clinician ever reviewing the images. Similarly, there were concerns around situations developing where a clinical service was dependent on AI functions that could not be replicated or performed by humans, or where human capabilities or clinical staff were not retained as a backup in case of AI failures. These concerns were mitigated considerably in processes where humans could provide ongoing review of AI decisions—where “everything eventually gets a human report” (M08)—or where human capacities and skills are purposefully maintained. This indicates the importance of *autonomous redundancy*: integrating intelligent functionality with other technical and safety systems, including low‐technology safety protections and controls, to maintain safety redundancy.

### 3.4 Epistemic sources of risk and resilience

Epistemic sources of risk and resilience arise from the ways that knowledge and ignorance are constructed in the development and operation of intelligent systems. Epistemic issues motivated a range of concerns and opportunities for participants.

#### 3.4.1 Learning lag and learning capacity

The potential for *learning lag* was a concern, where development and deployment activities could exceed the systems, resources and capacity available to analyze and learn from those activities, and the surprises they may generate. This was particularly the case due to the potential for healthcare AI to be rapidly and regularly updated, rendering prior knowledge of system functionality and risks quickly out of date. More traditional medical technologies—such as hip replacement prosthetic implants—have longer development lifecycles that accommodate extensive periods of testing and validation. As one regulator explained, with AI the “cycles are much shorter, potentially days. Or per patient, if it is learning from every patient exposure. The design of a device could be changing with every patient!” (R11). The potential for AI to be rapidly and regularly improved was perceived as one of its main benefits, with participants emphasizing “it's important that the algorithm learns over time” (T12). However, this needed to be balanced with the learning capacity of the organizational and regulatory system. This indicates the important of *learning capacity*, where the scale and speed of developmental and deployment activities are aligned with the capacity, systems and resources available to analyze, and learn from those activities.

#### 3.4.2 Operational disengagement and operational engagement

Participants were attuned to threats posed by *operational disengagement*, where there are limited or reduced efforts to gather data on, engage with and make use of insights drawn from the first‐hand experiences of people interacting with an intelligent system. This was highlighted both in terms of the need for detailed local knowledge of the clinical processes and contexts within which AI is being implemented, and in terms of practical knowledge of the capabilities, functions, and limits of an AI system itself. As one clinician forcefully argued, “if you don't have engaged and interested people on the frontline doing this work then it's bloody dangerous” (C04). Participants emphasized the importance of the contextual insight and knowledge of people interacting directly with an AI system, “because each institution has different machinery, different voltages, different methods of taking X‐rays, they have to have different systems for each place. So it has to be bespoke for that unit” (R22). This indicates the importance of *operational engagement*: systematic efforts to gather data on, engage with and make use of insights drawn from the first‐hand experiences of people deploying and interacting with intelligent systems.

#### 3.4.3 Insensitivity to experience and sensitivity to experience

There were concerns about *insensitivity to experience*—failures to anticipate, notice, or effectively explore the safety implications of events experienced during development, deployment, or operational use of intelligent systems. These issues were viewed as particularly challenging due to the absence of detailed guidance on how to identify and investigate AI‐related safety incidents or monitor system performance. Participants believed safety incidents and adverse outcomes associated with AI were at some point inevitable and needed to be carefully prepared for: “if there is an incident, it depends on how much pre‐figuring has been done… It's not surprising to have mistakes, but how prepared is the [hospital] Trust?” (C06). Processes for routinely auditing the outputs of AI were viewed as critical but underspecified, leaving potential gaps in monitoring and understanding where and why failures were occurring. Some clinicians indicated rules of thumb, to “put some safety checks in place, keep auditing 10% of images” (C04) on an ongoing basis. This indicates the importance of *sensitivity to experience*: capability to anticipate, notice, and effectively explore the safety implications of events experienced during development, testing, deployment, and operation of intelligent systems.

#### 3.4.4 Simulatory inattention and simulatory attention

There were broad‐based concerns about risks of *simulatory inattention*—the limited or minimal use of simulation to explore, train, test, and improve the behavior of intelligent systems. Simulation, in a wide variety of forms, was viewed as central to safely developing and implementing AI in healthcare. One area of considerable focus was the production and use of “synthetic” data: high‐fidelity simulated data that are digitally synthesized to faithfully replicate clinical relations in real patient data. Regulators were keen to see reliable synthetic data made readily available both to help train AI and to subsequently test the system, without posing any risk to the privacy of real patients. Another example regularly referred to was the importance of operating new AI systems in the background, separate to ongoing care processes, for instance acting as a “ghost reader” of medical images. This allows the clinical operation and performance of an AI system to be simulated but in relation to actual patient data in real‐world care settings. This indicates the importance of *simulatory attention*: the central and systematic use of different forms of simulation to explore, test, train, and improve the behavior of intelligent systems.

#### 3.4.5 Competitive secrecy and cooperative sharing

*Competitive secrecy* was a concern to many participants: constraints on creating or sharing safety data due to reluctance to disclose commercially sensitive information or reveal performance weaknesses, or confusion about data privacy. Commercial sensitivities posed challenges to sharing safety‐relevant information, particularly in relation to algorithms or data sets considered proprietary. As one regulator highlighted: “data being used by companies developing the product is not always open to academic validation and testing. Sometimes it is part of the business model, as proprietary data” (R15). Data sharing could also be impacted by caution and fear related to breaching privacy regulations by sharing patient‐related data for the purpose of identifying problems and improving algorithms: “these companies are of the opinion that they can't use data for [that] secondary purpose… it's going to make that aspect of the service difficult” (R09). Participants highlighted the need for innovative mechanisms to share data for safety improvement, “perhaps in a limited fashion to prescribed academic community who could assess and evaluate it… a half‐way house to opening up” (R15). This indicates the importance of enabling *cooperative sharing*, the readiness to create and share safety data while managing any risks of disclosing private or commercially sensitive information.

### 3.5 Cultural sources of risk and resilience

Cultural sources of risk and resilience arise from the collective values, beliefs, norms, and practices that surround and support intelligent systems. Cultural issues shaped a range of concerns and opportunities for participants.

#### 3.5.1 Performative production and performance authenticity

Concerns existed about *performative production*, where attention and activity focused on maximizing system performance on narrow publicly reported metrics which may not represent underlying quality, safety, or performance. This was highlighted in relation to testing AI performance in isolation from real‐world clinical applications, particularly in widely reported retrospective studies where AI is trained and tested on historical data. As one clinician AI developer explained: “Lots of AI systems are experimental, they show good performance on retrospective data in lab conditions. But the humans we see [as patients] are not humans in lab conditions” (C02). Another echoed, “I don't think they [retrospective studies] are very informative, when the algorithm is placed into real clinical practice you can expect it to be pretty different in those situations” (C10). Another emphasized “in retrospective studies it is too easy to fool yourself, to have your finger on the scale” (C01). There was widespread acknowledgment that clinical AI must be tested and assessed in relation to actual impact on clinical care in real‐world settings: “local practice and performance” (M08). This indicates the importance of *performance authenticity*: focusing attention and activities on a diverse portfolio of metrics which authentically indicate underlying quality, safety, and performance of an intelligent system.

#### 3.5.2 Functionality hype and functional prudence

Participants identified risks of *functionality hype*, where exaggerated claims regarding the functionality and capability of intelligent systems could misrepresent actual system capability and performance. Excessive claims for AI functionality were seen as common (Emanuel & Wachter, 2019). One regulator noted, “there are things that people claim AI will do and there is what it actually does today” (R15), while a healthcare manager described discussions with AI developers sometimes featured a degree “of ‘cloak and dagger’‐ness of what the solution does versus what it is regulated for” (M34). Exaggerating the capability of a system was viewed as hazardous as it could influence how people understand, deploy, and use it. One regulator explained, “Lots of issues come with people not really understanding what it can and can't do, partly as manufacturers over‐sell it” (R35). A clinician described the risk bluntly: “People may not be as careful” (C01). Honesty and clarity were essential, as one regulator emphasized: “how tech suppliers communicate what they can do has got to be honest and transparent and clear” (R36). This indicated the importance of *functional prudence*, where claims of system functionality and capability appropriately represent and are carefully calibrated to the actual functions that a system can effectively perform.

#### 3.5.3 Concern quashing and concern coaxing

Risk was identified in relation to *concern quashing*, where stakeholders are discouraged or disempowered in raising safety concerns, speaking up or challenging prevailing assumptions due to lack of support or fears of punitive responses. Many participants noted that assessing the risk of new AI systems is of limited value if the process does not openly engage with a diverse group of stakeholders. For example, one healthcare manager highlighted the importance of communication and collaboration in producing hazard logs required by NHS clinical risk management standards for health technologies (NHS Digital, 2018), emphasizing that producing the document itself was far less important than enabling open discussion about the risks of a new system with many stakeholders. Another participant noted the importance of regulators and clinicians remaining constructively skeptical and showing leadership by speaking up about AI safety concerns, emphasizing their role as “trying to catch the system out. We are here to protect patient safety. You have to have a healthy cynicism about these things” (R22). This indicated the importance of *concern coaxing*, where stakeholders are actively encouraged and empowered to raise safety concerns, speak up and challenge assumptions, and supported in doing so.

#### 3.5.4 Developmental disintegration and developmental integration

Participants identified risks of *developmental disintegration*, where norms and values privileged rapid development and experimental deployment of intelligent systems and deprioritized integrated and systematic safety oversight. Cultures of rapid development and local experimentation were viewed as a challenge for safety assurance. Some noted that excessive governance could stifle exploration: “If we did that for every system we evaluated it would be bunged up in committees for months” (M08). However, several managers noted that enthusiasm to explore and develop AI was not always aligned with appropriate risk controls: “The deployment that I had seen… had all the enthusiasm of research projects, but very little of the risk analysis and governance around them. That hadn't been thought of” (C25). One regulator emphasized: “The barrier to entry is very, very low with software. With hardware you need a factory, design drawings, materials—our systems can work with that. With software, you need a laptop and a sofa… it can be a GP sitting in surgery and writing an algorithm at lunchtime and using it in the afternoon” (R11). This indicates the need for *developmental integration*: norms, values, and practices that privilege the careful and integrated oversight of system design, development, deployment, and use of intelligent systems.

#### 3.5.5 Presumptive reliability and presumptive fallibility

There were concerns about risks arising from *presumptive reliability*: assumptions and beliefs that existing processes and practices are safe and can reliably accommodate intelligent systems with little additional support or monitoring. Some AI developers believed that there were few risks associated with AI because systems would be supervised by humans, indicating a presumption of safety and that human supervision was sufficient to mitigate any risk: “At this moment, the risks are quite minimal, no one is going to deploy AI without supervision” (T21). However, clinicians emphasized that while safety assurance processes existed in some areas of clinical practice—such as detecting errors through discrepancy reporting in radiology—this was not always the case. Likewise, meaningful measures of current levels of safety and reliability were not commonly available for many clinical processes. As such, participants believed it was important to develop and deploy AI based on a deep assumption of fallibility and failure, and that any new technologies and processes should be designed to accommodate this. As one regulator summarized: “No doubt it will miss things. That has to be built in” (R22). This indicated the importance of *presumptive fallibility*: assumptions and beliefs that current processes and practices are inherently fallible and can only reliably accommodate intelligent systems with extensive support and monitoring.

## 4 CONCLUSIONS: SOCIOTECHNICAL RISK AND RESILIENCE IN AIS

**FIGURE 4:**
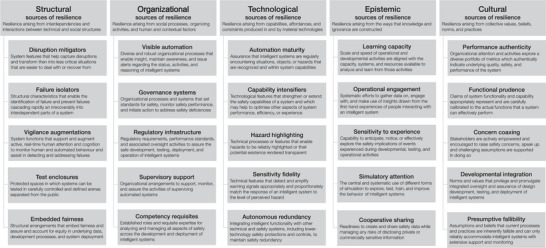
The structural, organizational, technological, epistemic and cultural (SOTEC) framework of the sociotechnical patterns of resilience associated with implementing clinical artificial intelligence.

The development and deployment of AI, and of AIS more generally, has the potential to bring considerable benefits to society—but realizing those benefits will require careful management of risk. The analysis presented here explores the sociotechnical nature of many of the fundamental sources of risk associated with developing, deploying, and using AI in healthcare. It also develops an initial characterization of the sociotechnical sources of resilience that may counter those risks. The analysis does this by refining and extending the SOTEC framework (Macrae, 2022) to provide a conceptual map of the risk landscape (Figure 4) which may be used to guide further research and focus regulatory attention, particularly in healthcare but also across other domains where intelligent systems are being deployed. This analysis also points to broader theoretical implications for the management of AI safety and risk, particularly regarding the safe integration of human and machine intelligence and the governance of risk and responsible innovation. These issues are considered in turn.

### 4.1 Characterizing and mapping sociotechnical sources of risk and resilience

The SOTEC analysis framework provides a way of characterizing and organizing the sociotechnical sources of risk associated with AIS. This study explored an array of AI risk management challenges in healthcare. In doing so, it extended the SOTEC framework in several important ways. In particular, two patterns of sociotechnical risk that were underdeveloped in the initial SOTEC framework were identified and elaborated. First, *embedded biases* are a critically important structural source of risk in healthcare AI where there is the potential to adversely impact large cohorts of patients by amplifying and perpetuating existing biases in the provision of care (Obermeyer et al., 2019). Second, *functionality hype* is a cultural source of risk important in healthcare settings where a nuanced and accurate understanding of the specific capabilities of AI are critical to safe integration with complex clinical practices (Nilsen et al., 2022). These sources of risk were serious concerns for healthcare participants in this study, and issues of AI bias and fairness (Challen et al., 2019; Lu et al., 2022) and the functionality and integration of AI (Coiera, 2019; Dixon, 2020) are emerging as some of the most urgent challenges in the field.

These additions extend the risk landscape characterized by SOTEC analysis and illustrate that the SOTEC framework—like any risk map—should be used as a developmental object of enquiry rather than a complete and final picture of risk. This is further emphasized by the other refinements made to the SOTEC analysis framework through this analysis. More broadly, these elaborations suggest that an array of “midrange” sources of AI risk have yet to be systematically characterized—or addressed by the regulatory community. The main focus of both research and regulatory attention to date has primarily been at two ends of a spectrum: work has either focused on granular technical issues associated with assurance and validation of individual AI technologies (Amodei et al., 2016; Esteva et al., 2019); or it has sought to develop high‐level principles and general guidance for ethical and safe use of AI (Jobin et al., 2019). The SOTEC analysis framework addresses the gap between “low‐level” technical assurance and “high‐level” ethical principles, enabling a midrange analysis of the sources of risk and resilience embedded in the practical, situated work of actually deploying and using AI in complex organizational settings, informed by foundational theories of sociotechnical risk (e.g., Perrow, 1999; Reason, 1997; Vaughan, 1996). To effectively manage and regulate the risks of AIS, much more research and regulatory attention will need to focus on such “midrange” sociotechnical analysis, both within healthcare (Larson, et al., 2021; Li et al., 2020; Nilsen et al., 2022; Smith et al., 2021) and across other domains. This is becoming increasingly urgent as new AI technologies, such as those based on large language models including OpenAI's ChatGPT (Thirunavukarasu et al., 2023), are rapidly being deployed and embedded in a range of societally‐critical systems.

### 4.2 Safely integrating human and machine intelligence

The analysis developed here has implications for how human and machine intelligence can be safely integrated in complex organizational settings, and how risk governance and regulatory processes can be designed to enable responsible innovation. First, developing this SOTEC framework in healthcare engages with many fundamental principles in the study of human factors and the safety sciences. The SOTEC framework is informed by a wide range of foundational theory, from Turner's (1978) exploration of the cultural incubation of disaster, to Perrow's (1984) analysis of the risks of structural complexity and coupling in organizations, to Reason's (1997) conceptualization of the latent organizational factors that contribute to failure, to Downer's (2011, 2024) theorization of epistemic accidents that emerge beyond the limits of formal knowledge. The SOTEC analysis developed here explored many of these sociotechnical patterns of risk in healthcare AI, but also speaks more broadly to wider debates about how to safely integrate human and machine intelligence.

One of these debates relates to the degree to which automation and machine intelligence either replaces, displaces, or transforms the cognitive and practical work required of humans (Lebovitz et al., 2022), and how automated systems should interact with and hand‐off work to humans (Young & Stanton, 2023). This challenge has animated the field for decades, importantly in Bainbridge's (Bainbridge, 1983) pioneering exploration of the “ironies” of automation: namely, that rather than removing or reducing the need for human involvement, the increasing complexity of automated systems actually demands equivalent increases in the sophistication and intensity of human oversight to ensure safety. These ironies persist in relation to AI (Endsley, 2023). The SOTEC analysis presented here contributes to these debates by identifying a set of sociotechnical patterns of resilience that seek to enhance the safe integration of human and machine intelligence and accommodate the increased cognitive and organizational demands that can be created by highly automated, autonomous, and intelligent systems.

Specifically, this SOTEC analysis highlights the importance of embedding intelligent technologies within *structures* and systems that are designed to extend rather than entirely replace human capabilities (“vigilance augmentation”). It emphasizes the importance of developing *organizational* processes and capacities that support the challenging human work of supervising and monitoring intelligent technologies (“supervisory support”). It points to the need for a *technological* approach that maintains low‐ or no‐technology processes to fall back on and perform critical functions if required (“autonomous redundancy”). It indicates the *epistemic* value of purposefully seeking out the insights and experiences of people interacting directly with intelligent technologies (“operational engagement”). And it highlights the importance of *cultural* premises and practices that actively engage with the inevitability of both human and technical failure, deploying intelligent technologies with that failure in mind (“presumptive fallibility”). Characterizing these sociotechnical patterns of resilience expands existing principles for the safe design of intelligent systems (Murphy & Woods, 2009) and indicates the types of interconnected processes that will be required to ensure the safe deployment and use of intelligent systems. It also emphasizes the value of continuing to refine systematic and integrative methods for human factors and safety analysis (Waterson, 2020), as well as the critical importance of enabling genuinely collaborative and mutually supportive interaction in human–machine teams (Sujan et al., 2019).

### 4.3 Regulation, responsible innovation, and governing resilient intelligent systems

The SOTEC analysis developed here also contributes to emerging debates on the regulation of risk and governance of responsible innovation in relation to AI, in healthcare and more widely. One of the most urgent challenges in the regulation and governance of AI concerns how to safely deploy AI in organizationally complex and societally consequential settings, and how to subsequently monitor and govern ongoing performance. AI technologies pose particular regulatory and governance challenges as initial deployment is often combined with lengthy processes of validation, testing, and iterative refinement in real‐world operational settings (Wiens et al., 2019), followed by regular and ongoing changes as operational data are used to further train and refine algorithmic performance over time. This complicates the regulatory boundaries that typically distinguish technical development of a technology from its operational use, blurring the line between experimental innovation and large‐scale public‐facing deployment (Stilgoe, 2019).

Risk management challenges associated with the complicated “last mile” of AI implementation (Coiera, 2019) are the focus of the SOTEC analysis developed here. This therefore provides an initial map of the types of sociotechnical process that regulators and risk managers may need to engage with, particularly within newly emerging regulatory and governance strategies for AI. These include regulatory sandboxes (CQC, 2020; FCA, 2015), where regulators, developers, and users of AI work together to test and understand the safety of intelligent systems within real‐world contexts (Allen, 2019; Zetzsche et al., 2017), providing an ideal arena in which to explore sociotechnical sources of risk and resilience. It also includes algorithmic auditing (Falco et al., 2021; Liu et al., 2022), where the performance and impacts of algorithmic technologies are systematically reviewed through structured analysis in collaboration with the teams deploying and using AI technologies. This represents a governance process that could closely engage with sociotechnical sources of risk and resilience, beyond the technical performance of the algorithm itself.

More broadly, this SOTEC analysis reinforces the importance of expanding risk regulation and governance strategies to actively build “positive” capabilities for resilience and safety as well as mitigate “negative” sources of risk. A primary example of this is the need to build structures that actively enable and assure fairness in the deployment and use of intelligent systems. Enabling fairness requires more than simply monitoring and eliminating sources of bias (Abràmoff et al., 2023; Chen et al., 2023) and will depend on, for instance, establishing equity‐focused standards for the construction of data sets underlying intelligent systems (e.g., Arora et al., 2023). In addition, this highlights the importance of actively cultivating norms, principles, and cultures of responsible innovation within the social and governance practices that surround intelligent systems (e.g., Naughton et al., 2023), as well as building the sociotechnical infrastructure that enables responsible development and deployment of AIS (e.g., Winfield et al., 2021).

### 4.4 Toward safe and resilient AIS

This article analyzed the sociotechnical sources of risk and resilience in autonomous and intelligence systems, particularly as they relate to the development, deployment, and use of AI in healthcare. The participants in this study engaged with issues of risk and resilience in an integrated and interconnected way: sources of risk and sources of resilience were not easily separated. The ease with which participants moved between discussions of risk and resilience allowed an initial map of the “positive” and enabling characteristics of resilience to be developed, as well as a more detailed exploration of the sources of risk. This analysis reinforces a foundational principle in the field of risk and resilience: that any useful explanation of safety requires a coherent analysis of the sources of risk that may cause harm as well as the sources of resilience that protect against those sources of harm (Anderson et al., 2020; Lyng et al., 2022; Macrae, 2014). Safety emerges from this elision of risk and resilience. This analysis provides a further step toward understanding the sociotechnical sources of risk and resilience in the AIS that will increasingly need to be regulated, governed, and managed.
